# Gene signature for the prediction of the trajectories of sepsis-induced acute kidney injury

**DOI:** 10.1186/s13054-022-04234-3

**Published:** 2022-12-21

**Authors:** Zhongheng Zhang, Lin Chen, Huiheng Liu, Yujing Sun, Pengfei Shui, Jian Gao, Decong Wang, Huilin Jiang, Yanling Li, Kun Chen, Yucai Hong, CMAISE Consortium, Lifeng Xing, Senjun Jin, Jian Sun, Yi Yang, Xiaohong Jin, Min Yang, Chunmei Gui, Yingpu Yuan, Guangtao Dong, Weizhong Zeng, Jing Zeng, Guoxin Hu, Lujun Qiao, Jinhua Wang, Yonglin Xi, Nan Wang, Minmin Wang, Yan Teng, Junxia Hou, Qiaojie Bi, Gengsheng Zhang, Junru Dai

**Affiliations:** 1 Department of Emergency Medicine, Key Laboratory of Precision Medicine in Diagnosis and Monitoring Research of Zhejiang Province, Sir Run Run Shaw Hospital, Zhejiang University School of Medicine, Hangzhou, 310016 People’s Republic of China; 2 Department of Critical Care Medicine, Affiliated Jinhua Hospital, Zhejiang University School of Medicine, Jinhua, People’s Republic of China; 3 Emergency Department, Zhongshan Hospital of Xiamen University, Xiamen, Fujian People’s Republic of China; 4 Department of Emergency, People’s Hospital of Anji, Anji County, Zhejiang People’s Republic of China; 5 Department of Critical Medicine, Pi County Peoples Hospital, Chengdu, People’s Republic of China; 6 Emergency Department, The Second Affiliated Hospital of Guangzhou Medical University, Guangzhou, People’s Republic of China

**Keywords:** Sepsis, Acute kidney injury, Support vector machine, RNA-seq, Genetic algorithms

## Abstract

**Background:**

Acute kidney injury (AKI) is a common complication in sepsis. However, the trajectories of sepsis-induced AKI and their transcriptional profiles are not well characterized.

**Methods:**

Sepsis patients admitted to centres participating in Chinese Multi-omics Advances In Sepsis (CMAISE) from November 2020 to December 2021 were enrolled, and gene expression in peripheral blood mononuclear cells was measured on Day 1. The renal function trajectory was measured by the renal component of the SOFA score (SOFA_renal_) on Days 1 and 3. Transcriptional profiles on Day 1 were compared between these renal function trajectories, and a support vector machine (SVM) was developed to distinguish transient from persistent AKI.

**Results:**

A total of 172 sepsis patients were enrolled during the study period. The renal function trajectory was classified into four types: non-AKI (SOFA_renal_ = 0 on Days 1 and 3, *n* = 50), persistent AKI (SOFA_renal_ > 0 on Days 1 and 3, *n* = 62), transient AKI (SOFA_renal_ > 0 on Day 1 and SOFA_renal_ = 0 on Day 3, *n* = 50) and worsening AKI (SOFA_renal_ = 0 on Days 1 and SOFA_renal_ > 0 on Day 3, *n* = 10). The persistent AKI group showed severe organ dysfunction and prolonged requirements for organ support. The worsening AKI group showed the least organ dysfunction on day 1 but had higher serum lactate and prolonged use of vasopressors than the non-AKI and transient AKI groups. There were 2091 upregulated and 1,902 downregulated genes (adjusted *p* < 0.05) between the persistent and transient AKI groups, with enrichment in the plasma membrane complex, receptor complex, and T-cell receptor complex. A 43-gene SVM model was developed using the genetic algorithm, which showed significantly greater performance predicting persistent AKI than the model based on clinical variables in a holdout subset (AUC: 0.948 [0.912, 0.984] vs. 0.739 [0.648, 0.830]; *p* < 0.01 for Delong’s test).

**Conclusions:**

Our study identified four subtypes of sepsis-induced AKI based on kidney injury trajectories. The landscape of host response aberrations across these subtypes was characterized. An SVM model based on a gene signature was developed to predict renal function trajectories, and showed better performance than the clinical variable-based model. Future studies are warranted to validate the gene model in distinguishing persistent from transient AKI.

**Supplementary Information:**

The online version contains supplementary material available at 10.1186/s13054-022-04234-3.

## Take home message

The study identified four subtypes of sepsis-induced AKI based on the kidney injury trajectories. The landscape of the host response aberrations across these subtypes was characterized. An SVM model based on gene signature was developed to predict renal function trajectories, which showed higher performance than the clinical variable-based model in the holdout subset. Future studies are warranted to validate the gene model in distinguishing persistent from transient AKI.

## Background

Acute kidney injury (AKI) is a common complication of sepsis and a well-known risk factor for adverse clinical outcomes, including increased mortality, prolonged length of stay in the intensive care unit (ICU), and development of chronic kidney disease (CKD) [1, 2]. Strenuous efforts have been made for management of sepsis-induced AKI, aiming to reduce the risks of these adverse clinical outcomes. Kidney Disease: Improving Global Outcomes (KDIGO) suggests comprehensive interventions to improve AKI outcomes, including protocol-based management of haemodynamic and oxygenation parameters, energy intake of 20–30 kcal/kg/d, protein intake restriction, and monitoring of aminoglycoside drug levels [3, 4]. However, the effects of these interventions are less than satisfactory due to the heterogenous AKI population [3, 5, 6]. Responses to certain interventions can differ based on the cause of AKI. Thus, it would be better to explore AKI based on the underlying causes.

Sepsis is the consequence of uncontrolled inflammatory responses to infection, leading to multiple organ dysfunction. Since the kidney is one of the most frequently affected organs, sepsis-induced AKI has been extensively explored in the literature [7, 8]. Sepsis-induced AKI has been reported to follow different renal function trajectories [9]. KDIGO defines persistent AKI as renal dysfunction beyond 48 h from AKI onset; otherwise, AKI is considered transient [10]. The characteristics of these renal function trajectories have been described in the literature [9, 11, 12]. However, the current AKI definition criteria cannot fully capture AKI progression on subsequent days. There is evidence showing that the initial AKI severity has limited performance for predicting kidney disease progression [13, 14]. Furthermore, the KDIGO criteria involve 48 h or more to define persistent or transient AKI, and it is clinically relevant to explore whether it is feasible to predict the renal function trajectory as early as possible.

Quantification of more novel transcripts and non-coding RNAs is possible with the development of in-depth next-generation RNA sequencing (RNA-Seq) technology [15, 16]. Studies in other fields have shown that such novel transcripts assist in identifying more potential mechanisms driving disease development and improve the accuracy of subtype prediction [17]. However, it is unknown whether gene signatures can be developed to predict the renal function trajectory. In this study, we first classified trajectories of sepsis-induced AKI by the renal component of the SOFA score, and then, transcriptional profiles between different renal function trajectories were characterized. Finally, we developed a simplified support vector machine classifier to distinguish transient from persistent AKI with genes filtered by genetic algorithms. We hypothesized that gene signatures measured on Day 1 can accurately predict subsequent renal function trajectories.

## Methods

### Study setting and patient enrolment

This study was conducted under the Chinese Multi-omics Advances In Sepsis (CMAISE) consortium from November 2020 to December 2021, involving 17 Chinese hospitals. The study protocol was registered at Chinese Clinical Trial Registry (http://www.chictr.org.cn/; ChiCTR2000040446). The English version of the registration website is https://www.chictr.org.cn/enIndex.aspx. Patients were considered eligible if they met the Sepsis-3.0 criteria (suspected or documented infection plus acute increase in Sequential Organ Failure Assessment (SOFA) score > 2 points) on admission to the Emergency Department (ED) [18]. Subjects were excluded if they met one of the following criteria: (1) end-stage cirrhosis with Child‒Pugh C; (2) concomitant malignancy or autoimmune disease; (3) do-not-resuscitate order; (4) pregnancy; (5) sepsis onset > 48 h or treatment at other hospitals when presenting to CMAISE member hospitals; (6) immunosuppression, such as long-term use of immunosuppressive agents, chemotherapy, corticosteroids, radiotherapy or HIV infection; (7) acute myocardial infarction and/or pulmonary embolism; and (8) preexisting chronic kidney disease (CKD). CKD was defined as the presence of one or more kidney damage markers for over 3 months: albuminuria (albumin excretion rate > 30 mg/24 h; albumin-to-creatinine ratio > 30 mg/g [> 3 mg/mmol]); urine sediment abnormality; electrolyte and other abnormality due to tubular disorders; abnormalities detected by histology; structural abnormalities detected by imaging; history of kidney transplantation; or GFR < 60 ml/min/1.73 m^2^. The study was approved by the ethics committee of Sir Run Run Shaw Hospital (approval number: 20201014-39). Informed consent was obtained from the patients or their next of kin surrogates.

### Variables and definitions

Baseline variables such as age, sex, height, and weight were recorded on admission. Laboratory variables including C-reactive protein, serum creatinine, urine output, procalcitonin, and coagulation profiles were obtained on Days 1, 3, and 5. In contrast to the conventional AKI definition, our study defined renal function trajectories by the renal component of the SOFA score (SOFA_renal_). Conventional definitions of AKI, such as the RIFLE, AKIN, or KDIGO criteria, are suitable for identifying AKI on admission but not for measuring the trajectory of changes in kidney function on consecutive days [19]. For instance, these criteria require baseline creatinine in the prior 2 or 7 days, which are not available for most emergency patients [20]. Furthermore, AKI grading requires > 24 h to define the severity of renal injury, which is not easy to use for trajectory definition.

The included subjects were classified into four types according to renal function trajectory. Cases without the development of AKI (SOFA_renal_ = 0) from Day 1 to Day 3 were considered as “non-AKI”. Those with SOFA_renal_ > 0 on Day 1 and SOFA_renal_ = 0 on Day 3 were considered transient AKI; those with SOFA_renal_ = 0 on Day 1 and SOFA_renal_ > 0 on Day 3 were considered worsening AKI, and those with SOFA_renal_ > 0 on Days 1 and 3 were considered persistent AKI.

### RNA-seq quantifications

Blood samples were obtained on Day 1, and peripheral blood mononuclear cells (PBMCs) were isolated by using density-gradient centrifugation according to a standard protocol. Total RNA was extracted and purified using TRIzol reagent (Invitrogen, Carlsbad, CA, USA) following the manufacturer's procedure and then stored at − 80 °C. All samples were sent for library preparation and gene expression quantification (LC-Bio Technologies (Hangzhou) Co., LTD.). Differential gene expression analysis was performed by using the DESeq2 pipeline [21]. Genes with less than 100 counts in all samples were removed. We calculated a variance stabilizing transformation (VST) from the fitted dispersion-mean relation(s) and then transformed the count data (normalized by division by the size factors or normalization factors), yielding a matrix of values that are now approximately homoscedastic (having constant variance along the range of mean values). The transformation also normalizes concerning library size [22]. Batch effects that might result from different institutions were removed using a design matrix including a term describing the sample source. The function fitted a linear model to the data, including both batches and types of AKI, and then removed the component due to the batch effects. Differential gene expression between transient versus persistent AKI, as well as worsening versus non-AKI was visualized using volcano plots. To facilitate biological interpretations, GO term enrichment of over-expressed genes was assessed [23].

### Gene signature for prediction of renal function trajectory

A prediction model based on the transcriptomic profile was trained by using the genetic algorithm (GA). The purpose of developing the prediction model is (1) to identify important biomarkers at the transcriptome level to indicate future studies and (2) to develop a simplified model to predict AKI progression as early as possible. GAs are variable search procedures based on the principle of evolution by natural selection. The procedure operates by evolving sets of variables (chromosomes) that fit certain criteria from an initial random population via cycles of differential replication, recombination, and mutation of the fittest chromosomes. Accuracy was used as the metric for the fitness function, and an accuracy > 0.9 was the goal to stop evolution. A total of 1000 cycles of evolution were run to select the best-fit chromosome (Additional file 1: methods) [24]. SVM with C-classification was trained to distinguish persistent from transient AKI [25]. The radial basis exp(−gamma*|u−v|^2^) was used as the kernel. Two hyperparameters gamma and cost were tuned by the grid search method. The cost is the ‘C’-constant of the regularization term in the Lagrange formulation. Threefold cross-validation was employed to estimate accuracy for a given chromosome. This approach involves randomly dividing the set of observations into 3 groups, or folds, of approximately equal size. The first fold is treated as a validation set, and the method is fit on the remaining 2 folds. A representative model was developed by using a forward selection strategy (Additional file 1: methods).

An SVM was also developed based on clinical variables (Additional file 1: Table E1), which was then compared to the gene model in the holdout subset that was generated by random sampling with a 1:2 ratio.

### Statistical analysis

Clinical and laboratory variables were compared between sepsis-induced renal function trajectories using conventional statistical methods. The chi-square test was used to compare categorical data. Normality in data distributions was assessed using the Anderson‒Darling test [26]. Analysis of variance was employed for normally distributed numeric data, and the Kruskal‒Wallis rank sum test for nonnormally distributed data. All statistical analyses were performed in R (version 4.1.1).

## Results

### Study population and clinical characteristics

**Fig. 1:**
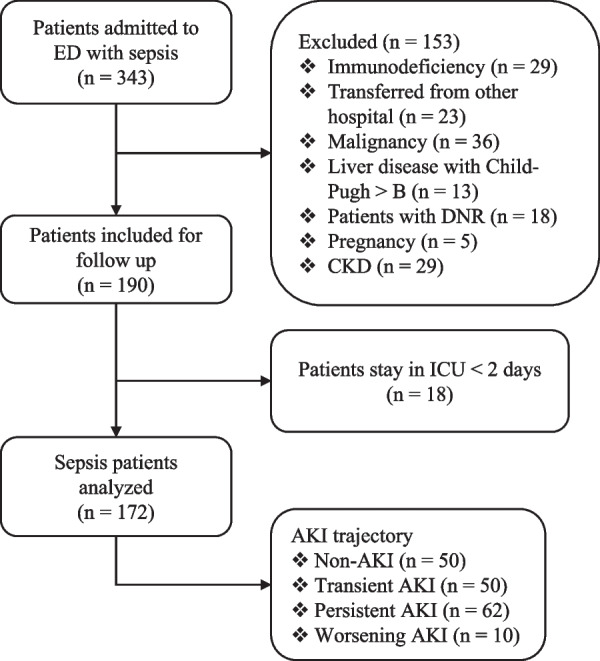
Flowchart of subject enrollment. ED = emergency department; CKD = chronic kidney disease; ICU = intensive care unit; AKI = acute kidney injury

**Fig. 2:**
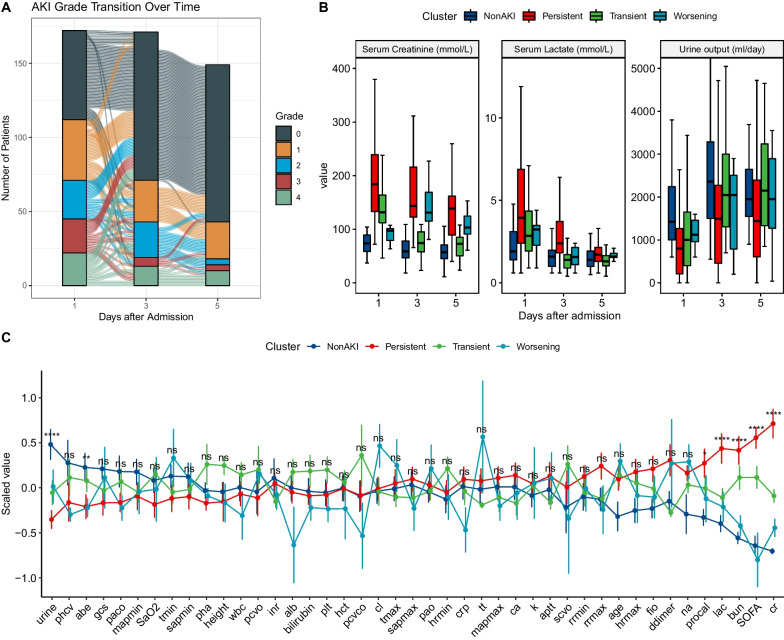
Sepsis-induced acute kidney injury trajectories. **A** Flowchart showing the transition of the severity of AKI as measured by renal component of the SOFA score. **B** Comparisons of serum lactate, creatinine and urine output between the four subtypes of sepsis-induced AKI. The boxplots describe the distribution of these variables. The error bar indicates the 95% confidence interval. The short line in the box indicates the median value. The four subtypes are denoted by filling colors. **C** Line plot showing the differences in clinical variables between the four subtypes. Statistical significance was annotated as: ns for > 0.05; * < 0.05; ** < 0.01, *** < 0.001, **** < 0.0001. AKI = acute kidney injury; scvo = central venous oxygen saturation; pcvo = central venous oxygen pressure; abe = actual base excess; pcvco = central venous carbon dioxide pressure; gcs = Glasgow coma scale; plt = platelet; sapmin = minimum systolic arterial pressure; cl = chloride; pao = arterial partial pressure of oxygen; pha = arterial pH; paco = arterial partial pressure of carbon dioxide; mapmin = minimum mean arterial pressure; phcv = central venous pH; rrmax = maximum respiratory rate; tmin = minimum temperature; hct = hematocrit; sapmax = maximum systolic arterial pressure; tt = thrombin time; ca = ionized calcium; fio = fraction of inspired oxygen; rrmin = minimum respiratory rate; inr = international normalized ratio; lac = lactate; SOFA = sequential organ failure assessment; alb = albumin

A total of 172 patients were included in the study (Fig. 1). Kidney injury severity grades were generally consistent between the renal component of the SOFA score and the RIFLE criteria (Additional file 1: Table E2). There were four subtypes of sepsis based on renal function trajectories: non-AKI (*n* = 50), persistent (*n* = 62), transient (*n* = 50) and worsening AKI (*n* = 10). The renal function trajectory measured by SOFA_renal_ was unstable across Days 1, 3, and 5 after hospital admission (Fig. 2A). There were more state transitions from Day 1 to 3 than from Day 3 to 5. Consistent with the definition for renal function trajectories, persistent AKI showed the highest serum creatinine and lowest urine output from Days 1 to 5 (Fig. 2B). The persistent AKI group exhibited greater severity of organ dysfunctions and prolonged requirement of organ support. Persistent AKI was related to the highest SOFA on Day 1 (9 [7, 11]; *p* < 0.001), longer days on mechanical ventilation (5.5 [0.25, 10.75] days; *p* = 0.003) and vasopressors (4.5 [1, 9]; *p* = 0.002). Although the worsening AKI group showed the least organ dysfunction on Day 1, this group had higher serum lactate levels and prolonged use of vasopressors than the non-AKI and transient AKI groups (Table 1), indicating delayed involvement of the kidney in this subtype.

**Table 1:** Comparison of clinical variables across different renal function trajectories

Variables	Total (*n* = 172)	Non-AKI (*n* = 50)	Persistent (*n* = 62)	Transient (*n* = 50)	Worsening (*n* = 10)	*p*
Age (years), Median (Q1, Q3)	72 (59.75, 81)	69.5 (52.25, 78.75)	73.5 (63, 81)	74 (62.5, 82)	72.5 (68.75, 81.75)	0.13
Sex, Male (%)	109 (63)	25 (50)	39 (63)	39 (78)	6 (60)	0.033
SOFA, Median (Q1, Q3)	8 (5, 10)	5.5 (4, 7)	9 (7.25, 11)	8 (6, 9)	4 (2.25, 7)	< 0.001
HR_max_ (/min), Mean ± SD	115.94 ± 22.31	110.3 ± 21.37	119.87 ± 24.81	117.1 ± 19.35	114 ± 21.14	0.149
SAP_min_ (mmHg), Mean ± SD	86.89 ± 18.89	89.22 ± 20.27	85.05 ± 20.85	86.55 ± 15.98	88.4 ± 11.64	0.702
*T*_max_ (℃), Median (Q1, Q3)	38 (37.1, 38.95)	37.85 (37.2, 39)	38 (36.82, 39.27)	38 (37.2, 38.5)	38.3 (37.62, 38.92)	0.774
Creatinine (mmol/L), Median (Q1, Q3)	123.4 (80.6, 174.5)	73.65 (58.6, 89)	184 (133.25, 239)	131.3 (112, 163.6)	97 (79.17, 101.07)	< 0.001
PaO_2_ (mmHg), Median (Q1, Q3)	91.3 (74.15, 113.93)	88 (73.75, 111)	87.2 (72.42, 111.5)	93.8 (75.15, 115.47)	94.5 (89.5, 118.65)	0.596
ABE (mmol/L), Median (Q1, Q3)	−4.6 (−7.9, −0.7)	−2 (−6.38, 1.55)	−5.9 (−10.28, −2.25)	−3.7 (−6.68, −0.43)	−5.05 (−6.97, −4.4)	0.007
BUN (mg/dl), Median (Q1, Q3)	9.94 (7.5, 14.5)	7.5 (5.7, 9.5)	13.48 (9.77, 17.12)	11.86 (8.55, 14.02)	8.13 (7.72, 8.8)	< 0.001
CRP (mg/dl), Median (Q1, Q3)	139.35 (55.7, 200)	143.6 (76.32, 183.58)	159.06 (53.94, 209.56)	125.85 (48.42, 203.1)	84.2 (32.4, 120.2)	0.491
Potassium (mmol/L), Mean ± SD	3.84 ± 0.66	3.79 ± 0.59	3.87 ± 0.67	3.86 ± 0.7	3.88 ± 0.82	0.91
Lactate (mmol/L), Median (Q1, Q3)	2.8 (1.62, 4.65)	1.91 (1.4, 3.1)	3.95 (2.4, 6.89)	2.85 (1.92, 4.35)	3.23 (2.28, 3.48)	< 0.001
Fluid intake (ml), Median (Q1, Q3)	2831 (1959, 4358.38)	2948.5 (2037.25, 4237.75)	2955 (2046, 4813)	2419 (1662, 4030)	2825 (2070.25, 4116.48)	0.238
Fluid output (ml), Median (Q1, Q3)	1400 (762, 2235)	1675 (1362.5, 2620)	1060 (560, 1905)	1140 (650, 1887)	1447.5 (1092.25, 1854.5)	0.004
Urine (ml), Median (Q1, Q3)	1050 (502.5, 1742.5)	1425.5 (1002.5, 2240)	800 (210, 1270)	1000 (400, 1650)	1122.5 (962.5, 1457.5)	< 0.001
Mortality, n (%)	19 (11)	2 (4)	14 (23)	3 (6)	0 (0)	0.008
MV days, Median (Q1, Q3)	3 (0, 7)	1 (0, 3.75)	5.5 (0.25, 10.75)	1.33 (0, 6)	0 (0, 5.75)	0.003
CRRT days, Median (Q1, Q3)	0 (0, 0)	0 (0, 0)	0 (0, 3.75)	0 (0, 0)	0 (0, 0)	< 0.001
Days on vasopressors, Median (Q1, Q3)	2 (0, 5.86)	1 (0, 3)	4.5 (1, 9)	2 (0, 5)	2.5 (0.5, 3)	0.002
Hospital days, Median (Q1, Q3)	13 (8.95, 21.04)	12.65 (8.77, 18.86)	11.76 (9.13, 22.51)	13.75 (8.47, 21.39)	16.08 (8.63, 19.5)	0.902

*Q1* first quartile, *Q3* third quartile, *HR* heart rate, *SAP* systolic arterial pressure, *SD* standard deviation, *BUN* blood urea nitrogen, *CRP* C-reactive protein, *MV* mechanical ventilation, *CRRT* continuous renal replacement therapy

### Transcriptomic profiles of renal function trajectories

**Fig. 3:**
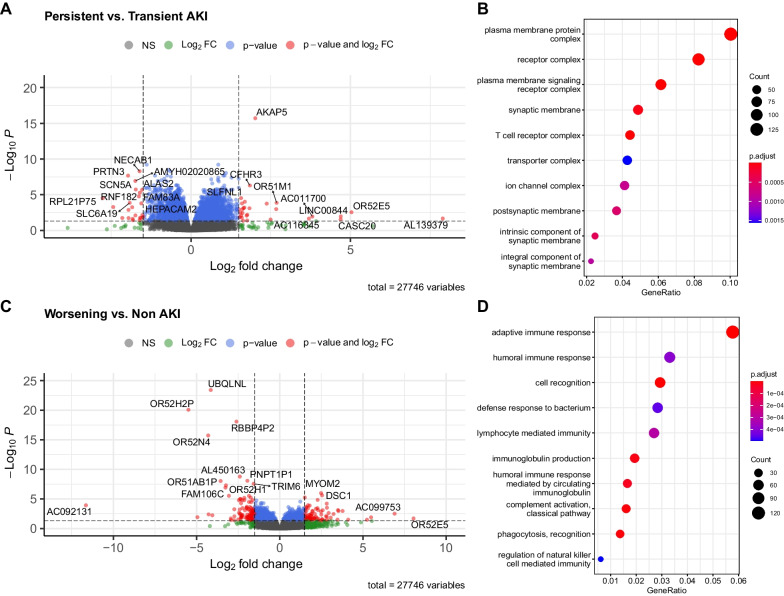
Differentially expressed genes between different trajectories of sepsis-induced AKI. **A** Volcano plot showing the differentially expressed genes between persistent and transient AKI groups. Genes with adjusted p value < 0.05 and log2 fold change > 1.5 were colored red and some example genes were labelled. **B** Enrichment of DEGs between persistent and transient AKI groups on GO terms by the overrepresentation method. **C** Volcano plot showing the differentially expressed genes between worsening and non-AKI groups. Genes with adjusted p value < 0.05 and log2 fold change > 1.5 were colored red and some example genes were labelled. **D** Enrichment of DEGs between worsening and non-AKI groups on GO terms by the overrepresentation method. AKI = acute kidney injury; FC = fold change; DEG = differential expressed gene; NS = non-significant

Differential gene expression analysis was performed between the worsening AKI versus non-AKI groups and the persistent versus transient AKI groups (Fig. 3). A total of 27,746 genes were filtered and tested for differential expression between the groups. There were 3,993 DEGs (adjusted *p* < 0.05) between the transient and persistent AKI groups, including 2091 upregulated and 1,902 downregulated genes (Fig. 3). The upregulated genes were enriched in biological pathways such as the plasma membrane complex, receptor complex, and T-cell receptor complex. There were 1,553 DEGs (adjusted *p* < 0.05) between the worsening and the non-AKI group, including 709 upregulated and 844 downregulated genes (Fig. 3). The upregulated genes were enriched in biological pathways such as adaptive immune response, humoral immune response, lymphocyte-mediated immunity, and immunoglobulin production (Fig. 3D).

### Genetic algorithm for developing an SVM to distinguish transient versus persistent AKI

**Fig. 4:**
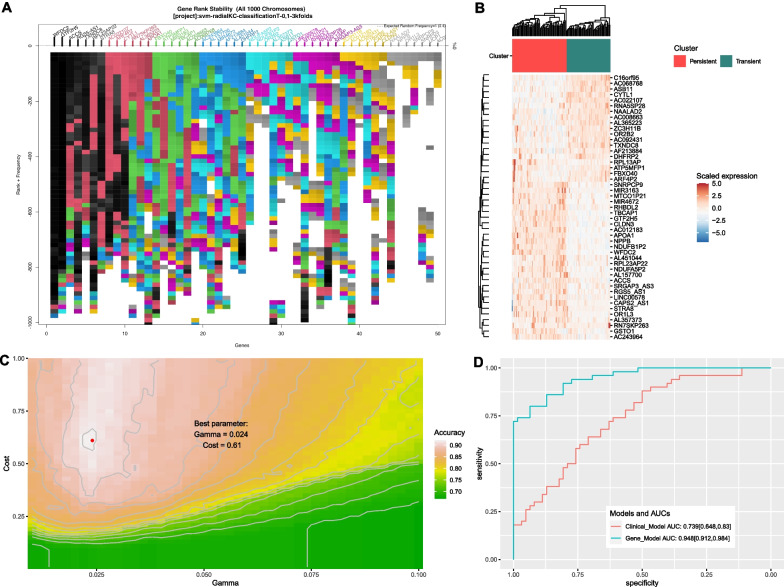
Development of a support vector machine model using genetic algorithms. **A** Stability of gene ranks over the 1000 evolution cycles. The plot shows the stability of the rank of the top 50 genes, which is designed to aid in the decision to stop or continue the process once the top ranked genes are stabilized. When genes have many changes in ranks, the plot show different colours; hence the rank of these genes is unstable. Commonly the top 2 “black” genes are stabilized quickly, in 50 to 200 solutions (evolutions), whereas low ranked “grey” genes would require many thousands of solutions to be stabilized. **B** heatmap plot showing the scaled gene expression abundance grouped by AKI groups. The genes displayed were selected by classical forward selection method, adding one gene at the time starting from the most frequent to the least frequent. **C** Hyperparameter tuning for training the SVM for the gene model. Contour plot shows the hyperparameter tuning process by the grid search method. Cost and gamma are two hyperparameters of the SVM model. The plot shows the accuracy of the SVM model (denoted by color) at each combination of cost (vertical axis) and gamma (horizontal axis), and the combination of the hyperparameters at the highest accuracy is used to train the final model. **D** Comparisons of the SVM models based on clinical variable and gene features. The gene model outperformed clinical model as indicated by significantly higher values of AUC

GA identified a 43-gene SVM model to distinguish persistent from transient AKI. The top-ranked genes were WFDC2, GTF2H5, ACCS, RGS5-AS1, TXNDC8, and RPL23AP22 (Additional file 1: Figure E1 to E3). Some non-coding RNAs with low expression were found to be important in predicting renal function trajectories, such as LINC00578, MIR3163, MIR4672, and AC068768 (Fig. 4A). Indeed, these selected genes were able to distinguish the two types of AKI in a heatmap plot (Fig. 4B). Hyperparameter tuning for the SVM showed that the best combination of gamma and cost was 0.024 and 0.61, respectively (Fig. 4C). We further fit an SVM based on clinical variables collected on Day 1 and found that these variables had moderate discriminating power to distinguish persistent versus transient AKI (AUC = 0.739; 95% CI: 0.648 to 0.830), which was significantly lower than the gene model (AUC = 0.948; 95% CI: 0.912 to 0.984). The model performance was evaluated using the holdout subset of data.

## Discussion

Our study describes the transcriptional landscape of different types of sepsis-induced renal function trajectories. Four subtypes of sepsis were identified according to the renal function trajectory: non-AKI, transient, persistent, and worsening AKI. Persistent AKI was the most critically ill group, as represented by the highest SOFA score and prolonged use of MV and vasopressors. There were hundreds to thousands of DEGs between these subtypes and pathways involving the adaptive immune response, humoral immune response, and lymphocyte-mediated immunity might explain the development of different renal function trajectories. We further developed SVM models comprising clinical or gene features, with features selected by genetic algorithms. The results showed that the clinical model had moderate discriminating power to distinguish persistent from transient AKI; in contrast, the gene signature model showed high accuracy.

The worsening subtype of AKI described in our study has not yet been formally defined in the consensus report of Acute Disease Quality Initiative (ADQI) 16 Workgroup [10]. This subtype involved normal renal function on admission but declining kidney function on the following days. Although this subtype comprised a minority of the sepsis population, important clinical implications were noted. Compared to the non-AKI group (i.e. both showed normal renal function on admission), the worsening AKI group had prolonged use of vasopressors, higher initial lactate levels, and longer hospital length of stay. Interestingly, the worsening AKI group showed remarkable host response aberrations on Day 1 compared to those without AKI. There were 709 upregulated and 844 downregulated genes compared with the non-AKI group. Pathways involving these DEGs are potential targets for the prevention of AKI development. DSCAM was significantly upregulated in the worsening group (log2FC = 5.24; adjusted *p* = 0.034). This gene has been found to mediate activation of MAPK8 and MAP kinase p38 [27, 28]. Consistent with our findings, p38 MAPK is involved in the development of sepsis-related multiple organ failure, including AKI [29–31]. It would be reasonable to hypothesize that inhibition of this pathway may protect against AKI. More importantly, there is sufficient time to implement preventive measures during a hospital stay. The “worsening” group had the lowest mortality rate and never required CRRT. This finding can be explained by the small sample size of this group, and the mortality or CRRT rate comparisons are subject to random variation.

Clinical and transcriptional alterations between persistent and transient AKI have been explored in the literature. Uhel F and colleagues compared transient and persistent AKI in a large cohort of sepsis [9]. Consistent with our findings, the persistent group had higher disease severity scores. However, minimal differences in transcriptional alterations between transient and persistent AKI were found, while our study identified more DEGs. Most likely, the advantages of RNA-Seq over microarray-based RNA quantification assisted us in identifying more biomarkers to distinguish between persistent and transient AKI. These advantages include the ability to detect novel transcripts (such as AC068768 and AC022107), lower noise signals, increased sensitivity in detecting differential expression, and the ability to quantify a large dynamic range of expression levels [32–34]. A machine learning model has been developed for the prediction of persistent or transient AKI using clinical data alone [11, 12]; the model performance was moderate, with an AUC below 0.80, which was consistent with our study. Nevertheless, the gene model was able to increase accuracy by a large magnitude owing to the sensitivity of RNA-Seq to identify novel and lowly expressed genes.

Several limitations must be acknowledged in the study. First, the study population was recruited from multiple centres in China, and there were potential batch effects in the RNA quantification performed. Regardless, we removed the batch effects with regression models, thereby minimizing the impact of such unwanted effects. Second, although our gene model showed high discriminating power in predicting persistent versus transient AKI, the model was not externally validated and was still subject to model overfitting. However, the study did identify many novel DEGs, which provided a more comprehensive transcriptional landscape for future mechanistic studies of sepsis-induced AKI. Third, prior measurement of kidney function may not be performed for some patients, making differentiation between CKD and AKI challenging. However, prior measurement of renal function was not carried out for only 5 patients, and only 2 of them showed elevated renal function on admission. Thus, we believe that the bias caused by the lack of prior renal function measurements in the study was minimal. Finally, the sample size of the worsening AKI group was relatively small compared with the other subtypes, limiting further in-depth characterization of this subgroup. Because AKI onset was delayed in this group, there is an opportunity to take measures to prevent AKI occurrence.

## Conclusions

Our study identified four subtypes of sepsis-induced AKI based on the renal function trajectory. The landscape of host response aberrations across these subtypes was characterized. An SVM model based on a gene signature was developed to predict renal function trajectories, which showed higher performance than the clinical variable-based model in the holdout subset. Future studies are warranted to validate the gene signature in distinguishing persistent from transient AKI.

## Supplementary Information

**Additional file 1.** Supplemental materials, figures and tables.

## Data Availability

Data are available upon formal approval in the National Genomic Data Center (https://ngdc.cncb.ac.cn/bioproject/browse/PRJCA006118).

## Abbreviations

SVM: Support vector machine
CMAISE: Chinese Multi-omics Advances In Sepsis
ICU: Invasive care unit
ED: Emergency department
HIV: Human immunodeficiency virus
PBMC: Peripheral blood mononuclear cells
AKI: Acute kidney injury
DEG: Differentially expressed gene
GA: Genetic algorithm
VST: Variance stabilizing transformation
