# Supplementary material for: Gene signature for the prediction of the trajectories of sepsis-induced acute kidney injury
Source: Crit Care. 2022 Dec 21;26:398. doi: 10.1186/s13054-022-04234-3 (PMC9773539; doi:10.1186/s13054-022-04234-3)
Supplement: Supplementary file 1 — Additional file 1. Supplemental materials, figures and tables. [file 13054_2022_4234_MOESM1_ESM.docx]

Electronic supplemental material for

Gene signature for the prediction of the trajectories of sepsis-induced acute kidney injury

Table of Contents

[Methods 2](#_Toc98246821)

[Blood sample preparation 2](#_Toc98246822)

[Bulk RNA-seq library preparation and quantification 3](#_Toc98246823)

[Genetic algorithm to filter candidate genes to predict cluster membership 3](#_Toc98246824)

[Support vector machine 4](#_Toc98246825)

[Development of a representative model 5](#_Toc98246826)

[Table E1. Variables used to develop the support vector machine 5](#_Toc98246827)

[Results 10](#_Toc98246828)

[Figure E1. Number of top ranked genes used by the models. 10](#_Toc98246829)

[Figure E2. Gene Frequency. 11](#_Toc98246830)

[Figure E3. Rank index of genes selected by the GA. 12](#_Toc98246831)

[Reference: 13](#_Toc98246832)

# Methods

## Blood sample preparation

Two EDTA tubes of 2 ml volume were used to collect peripheral blood samples on day 1, 3 and 5 after hospital admission and processed within 6 hours. Patients who were discharged/died before day 3 or 5 could not provide blood sample on these days.

1. Blood samples were transferred to centrifugation tubes and centrifugated at 2000 rpm for 5 min. A total of 1.5 ml supernatant was aspirated and stored at -80℃.
2. The remaining serum was removed.
3. 1 ml PBS was added to the centrifugation tube.
4. Mix the PBS and cells obtained from above steps and slowly moved to a tube filled with 2 ml Lymphocyte isolation solution.
5. Centrifugate at 2000 rpm for 20 min and the PBMC layer was aspirated.
6. The aspirated cells were put into centrifugation tube, centrifugate at 10000g for 3 min.
7. 250 ul PBS and 750 ul Trizol were added to the tube and stored at -80℃.

The RNA amount and purity of each sample was quantified using NanoDrop ND-1000 (NanoDrop, Wilmington, DE, USA). The RNA integrity was assessed by Agilent 2100 with RIN number >7.0.

## Bulk RNA-seq library preparation and quantification

Peripheral blood mononuclear cells (PBMC) were isolated by using density-gradient centrifugation as per standard protocol. Total RNA was extracted and purified using TRIzol reagent (Invitrogen, Carlsbad, CA, USA) following the manufacturer's procedure and were then stored at -80℃. Then all samples were sent for library preparation and gene expression quantification (LC-BioTechnologies (Hangzhou) Co., LTD.). The RNAs were reverse-transcribed to cDNA following ribosomal RNAs removal. U-labeled double-stranded DNAs was then synthesized with E. coli DNA polymerase I, RNase H and dUTP. The fragments were ligated with single-or dual-index adapters, and a size selection assay was performed with AMPureXP beads. U-labeled double-stranded DNAs were treated with heat-labile UDG enzyme. The ligated products were amplified with PCR under pre-established conditions. The average insert size for the final cDNA library was 300 bp (±50 bp). Finally, we performed paired-end sequencing on an Illumina NovaSeq™ 6000 following the vendor's recommended protocol.

The reads containing adapter contamination, low-quality bases and undetermined bases were removed using cutadapt-1.9 ([cutadapt.readthedocs.io/en/stable/](http://cutadapt.readthedocs.io/en/stable/)) [1]. Then sequence quality was verified using FastQC v0.10.1 ([www.bioinformatics.babraham.ac.uk/projects/fastqc/](http://www.bioinformatics.babraham.ac.uk/projects/fastqc/)). We used HISAT2-2.0.4 to map reads to the genome of *Homo sapiens* obtained from the Ensembl v96 database[2]. The mapped reads of each sample were assembled using StringTie-1.3.4 with default parameters[3]. Then, the Gffcompare tool was used to compare and merge different gene annotations [4]. After the final transcriptome was generated, the expression level of the RNAs were determined using StringTie to obtain FPKM values.

## Genetic algorithm to filter candidate genes to predict cluster membership

Genetic Algorithms (GAs) are variable search procedures based on the principle of evolution by natural selection. The procedure works by evolving sets of variables (chromosomes) that fit certain criteria from an initial random population via cycles of differential replication, recombination and mutation of the fittest chromosomes. In the study, we employed the

GALGO pipeline for gene filtering [5]:

Stage 1: The procedure initially creates a number of random gene sets (chromosomes). These variable sets form a population of chromosomes (niche). Each chromosome contains 10 random genes in our study.

Stage 2: Each chromosome in the population is evaluated for its ability to predict the group membership of each sample in the dataset. This is achieved by training a statistical model. The GA tests the accuracy of the prediction and assigns a score to each chromosome that is proportional to the accuracy resulted in the fitness function. Because the number of persistent and transient AKI are balanced, we used accuracy as the fitness function. Accuracy is calculated by dividing the correctly predicted sample by the total sample.

Stage 3: When a chromosome has a score higher then a predefined value (accuracy > 90%), this chromosome is selected and the procedure stops; otherwise, the procedure continues to stage 4.

Stage 4: The population of chromosomes is replicated. Chromosomes with a higher fitness score will generate a more numerous offspring.

Stage 5: The genetic information contained in the replicated parent chromosomes is combined through genetic crossover. Two randomly selected parent chromosomes are used to create two new chromosomes. This crossover mechanism allows a better exploration of possible solutions recombining good chromosomes.

Stage 6: Mutations are then introduced in the chromosome randomly. These mutations substitute new genes in the chromosomes.

Stage 7: The process is repeated from stage 2 until an accurate chromosome is obtained (accuracy > 90%). The cycle of replication (stage 4), genetic cross‐over (stage 5) and mutations (stage 6) is called generation.

The above steps constitute a cycle of evolution, the maximum number of generations is set to 200 in our study. If the maximum number of generations are reached but the accuracy is < 90%, this cycle is denoted as “no solution”. The chromosome with the highest accuracy is adopted. A total number of 1000 evolution cycles were implemented in the study.

Three-fold cross validation was employed to estimate the accuracy of a chromosome. This approach involves randomly dividing the set of observations into 3 groups, or folds, of approximately equal size. The first fold is treated as a validation set, and the method is fit on the remaining 2 folds (Figure 1).

## Support vector machine

Suppose that we plot every sample in a convenient plane distinguishing sample class. In support vector machines (SVM) the convenient plane is called kernel function and its purpose is to transform the data into a higher dimensional space that allows a better and easier separation[6]. For classification purposes, the best line that separate classes is that whose distance between the line and the closest samples within classes are maximum (because it allows the highest margin between those points). C-classification was set as the svm type as the task of the model was to classify persistent versus transient AKI. The kernel used in training and predicting is the radial basis exp(-gamma*|u-v|^2). The gamma is the hyperparameter to be tuned. Another hyperparameter was the cost of constraints violation, which is the ‘C’-constant of the regularization term in the Lagrange formulation. Grid search method was employed to obtain the best combination of the parameters.

## Development of a representative model

The above procedure produced a number of chromosomes with predefined accuracy (> 90%) in predicting transient versus persistent AKI. For the ease of clinical utility, there is a need to

develop a single model that is, to some extent, representative of the population. The simpler strategy to follow is to use the frequency of genes in the population of chromosomes as criteria for inclusion in a forward selection strategy. The model of choice will be the one with the highest classification accuracy and the lower number of genes. The GALGO pipeline also stores alternative models with similar accuracy (accuracy within 99% of the highest accuracy) and larger number of genes. This strategy ensures that the most represented genes in the population of chromosomes are included in a single summary model. This procedure should be applied to the population of chromosomes generated by the above GA search.

## Table E1. Clinical variables used to develop the support vector machine

|  | Category | Variable names | Note |
| --- | --- | --- | --- |
| 1 | Demographics | Cluster | Sepsis immune cluster obtained by k-means clustering |
| 2 | Demographics | age | In years |
| 3 | Demographics | sex | Male/female |
| 4 | Demographics | height | cm |
| 5 | Demographics | weight | Kg |
| 6 | Comorbidity | diabete | Diabetes mellitus |
| 7 | Comorbidity | hyperten | Hypertension |
| 8 | Comorbidity | myoinfarc | Myocardial infarction |
| 9 | Comorbidity | cardiofailure | Cardiac failure |
| 10 | Comorbidity | cerebrovasc | Cerebrovascular disease |
| 11 | Comorbidity | dementia | Dementia |
| 12 | Comorbidity | copd | COPD: Patients with an FEV1/FVC post brochodilation <70% with consistent symptom were diagnosed with COPD |
| 13 | Comorbidity | paralysis |  |
| 14 | Others | Days | The day on hospital admission was day 1. |
| 15 | Vital Signs | hrmax | Maximum heart rate recorded on nursing chart on the study day |
| 16 | Vital Signs | hrmin | Minimum heart rate recorded on nursing chart on the study day |
| 17 | Vital Signs | mapmax | Maximum mean arterial pressure recorded on nursing chart on the study day |
| 18 | Vital Signs | mapmin | Minimum mean arterial pressure recorded on nursing chart on the study day |
| 19 | Vital Signs | sapmax | Maximum systolic arterial pressure recorded on nursing chart on the study day |
| 20 | Vital Signs | sapmin | Minimum systolic arterial pressure recorded on nursing chart on the study day |
| 21 | Vital Signs | rrmax | Maximum respiratory rate recorded on nursing chart on the study day |
| 22 | Vital Signs | rrmin | Minimum respiratory rate recorded on nursing chart on the study day |
| 23 | Vital Signs | tmax | Maximum temperature recorded on nursing chart on the study day |
| 24 | Vital Signs | tmin | Minimum temperature recorded on nursing chart on the study day |
| 25 | Vital Signs | mv | Use of mechanical ventilation on the study day |
| 26 | Treatment | crrt | Use of continuous renal replacement therapy on the study day |
| 27 | Treatment | gcs | Glasgow comma scale |
| 28 | Treatment | lac | Serum lactate |
| 29 | Laboratory findings | k | Potassium |
| 30 | Laboratory findings | na | Sodium |
| 31 | Laboratory findings | cl | Chloride |
| 32 | Laboratory findings | ca | Total Calcium |
| 33 | Laboratory findings | pha | Arterial pH |
| 34 | Laboratory findings | paco | PaCO2 |
| 35 | Laboratory findings | pao | PaO2 |
| 36 | Laboratory findings | abe | ABE |
| 37 | Laboratory findings | fio | FiO2 |
| 38 | Laboratory findings | SaO2 | SaO2 |
| 39 | Laboratory findings | procal | Procalcitonin |
| 40 | Laboratory findings | phcv | Central venous pH |
| 41 | Laboratory findings | pcvco | Central venous CO2 |
| 42 | Laboratory findings | pcvo | Central venous O2 |
| 43 | Laboratory findings | scvo | Central venous O2 saturation |
| 44 | Laboratory findings | bun | BUN |
| 45 | Laboratory findings | alb | Albumin |
| 46 | Laboratory findings | cr | Serum creatinine |
| 47 | Laboratory findings | bilirubin | Total bilirubin |
| 48 | Laboratory findings | crp | C-reactive protein |
| 49 | Laboratory findings | wbc | WBC ($\times{10}^{9}$/L) |
| 50 | Laboratory findings | hct | HCT |
| 51 | Laboratory findings | plt | Platelet count ($\times{10}^{9}$/L) |
| 52 | Laboratory findings | inr | International normalized ratio |
| 53 | Laboratory findings | aptt | APTT |
| 54 | Laboratory findings | tt | TT |
| 55 | Laboratory findings | ddimer | D-Dimer |
| 56 | Laboratory findings | urine | Urine output ml/Day |
| 57 | Laboratory findings | sofa_vaso | Circulatory component of SOFA score |
| 58 | Scores | SOFA | SOFA score |

# Results

## Figure E1. Number of top ranked genes used by the models.

The number of genes present in all chromosomes and the number of top‐genes needed to cover fractions of them. This could help to decide how many genes to select covering more or less genes in chromosomes as criteria. For instance, the top 405 genes are the 25% of all different genes present in chromosomes. This is because these genes are highly repetitive in chromosomes.

## Figure E2. Gene Frequency.

Left axis shows the number of times the gene has been present in chromosomes. Right axis marks the corresponding percentage relative to the total number of chromosomes. Horizontal axis shows the value of the gene in the chromosome. Only the first “colour section” is labelled with their corresponding gene names.

## Figure E3. Rank index of genes selected by the GA.

This plot shows the ranks of all genes (horizontal axis) and their frequency in log scale (in vertical axis) to highlights small frequencies and compact high frequencies.

## Table E2. cross-tabulation of renal SOFA score and RIFLE stages on day 1

|  | 0 | 1 | 2 | 3 | 4 |
| --- | --- | --- | --- | --- | --- |
| None | 58 | 1 | 1 | 0 | 0 |
| Risk | 2 | 32 | 4 | 1 | 0 |
| Injury | 0 | 8 | 20 | 7 | 2 |
| Failure | 0 | 0 | 0 | 15 | 20 |
| Total | 60 | 41 | 26 | 23 | 22 |

# Reference:

1. Kechin A, Boyarskikh U, Kel A, Filipenko M. cutPrimers: A New Tool for Accurate Cutting of Primers from Reads of Targeted Next Generation Sequencing. J Comput Biol. 2017;24:1138–43.

2. Kim D, Langmead B, Salzberg SL. HISAT: a fast spliced aligner with low memory requirements. Nat Methods. 2015;12:357–60.

3. Pertea M, Pertea GM, Antonescu CM, Chang T-C, Mendell JT, Salzberg SL. StringTie enables improved reconstruction of a transcriptome from RNA-seq reads. Nat Biotechnol. 2015;33:290–5.

4. Pertea G, Pertea M. GFF Utilities: GffRead and GffCompare [version 1; peer review: 3 approved]. F1000Research [Internet]. 2020;9. Available from: https://f1000research.com/articles/9-304/v1

5. Trevino V, Falciani F. GALGO: an R package for multivariate variable selection using genetic algorithms. Bioinformatics. 2006;22:1154–6.

6. Ressom HW, Varghese RS, Zhang Z, Xuan J, Clarke R. Classification algorithms for phenotype prediction in genomics and proteomics. Front Biosci. 2008;13:691–708.
